# Preliminary Study on Electrophysiological Changes After Cellular Autograft in Age-Related Macular Degeneration

**DOI:** 10.1097/MD.0000000000000355

**Published:** 2014-12-02

**Authors:** Paolo Giuseppe Limoli, Enzo Maria Vingolo, Marco Ulisses Morales, Marcella Nebbioso, Celeste Limoli

**Affiliations:** From the Low Vision Research of Milan (PGL, CL), Milan; Department of Ophthalmology (EMV), A. Fiorini Hospital, Terracina; Polo Pontino (EMV); CenterVue (MUM), Padova; and Department of Sense Organs (MN), Faculty of Medicine and Odontology, Sapienza University of Rome, Rome, Italy.

## Abstract

Evolving atrophic macular degeneration represents at least 80% of all macular degenerations and is currently without a standardized care. Autologous fat transplantation efficacy was demonstrated by several studies, as these cells are able to produce growth factors. The aim of the work was to demonstrate possible therapeutic effect of the joined suprachoroidal graft of adipocytes, adipose-derived stem cells (ADSCs) in stromal vascular fractions (SVFs) of adipose tissue, and platelet-rich plasma (PRP).

Twelve eyes in 12 dry age-related macular degeneration (AMD) patients, aged 71.25 (SD ± 6.8) between 62 and 80 years, were analyzed. A complete ocular evaluation was performed using best corrected visual acuity (BCVA), retinographic analysis, spectral-domain optical coherence tomography, microperimetry, computerized visual field, and standard electroretinogram (ERG). Each eye received a cell in graft between choroid and sclera of mature fat cells and ADSCs in SVF enriched with PRP by means of the variant second Limoli (Limoli retinal restoration technique [LRRT]). In order to test if the differences pre- and post-treatment were significant, the Wilcoxon signed-rank test has been performed.

Adverse effects were not reported in the patients. After surgery with LRRT, the most significant increase in the ERG values was recorded by scotopic rod-ERG (answer coming from the rods), from 41.26 to 60.83 μV with an average increase of 47.44% highly significant (*P* < 0.05). Moderately significant was the one recorded by scotopic maximal ERG (answer coming from the rods and cones), from 112.22 to 129.68 μV with an average increase of 15.56% (*P* < 0.1).

Cell-mediated therapy based on growth factors used appears interesting because it can improve the retinal functionality responses in the short term. The ERG could, therefore, be used to monitor the effect of cell-mediated regenerative therapies.

## INTRODUCTION

Evolving atrophic macular degeneration represents at least 80% of all macular degenerations and is currently without a standardized care. However, since the 1990s, a series of studies using growth factors to block or slow retinal atrophy has begun.^1–9^ The eredodistrophic guinea pigs allow to note the photoreceptor survival promotion, counting the nuclear layer thickness after intravitreal injection of growth factor.^10,11^

Application of therapy for regenerative purpose in humans is hampered by the rapid half-life of these factors that would require intraocular injections at least every 2 months, with the potential implications that it entail. In fact, some serious side effects have been noted including macular hemorrhages, vitreoretinal interface alterations, intravitreal hemorrhages, retinal detachment, cataract, proliferative vitreoretinopathy, and endophthalmitis.^1,12,13^

One possible source of autologous growth factors is represented by adipose tissue.^14–17^

Autologous fat transplantation efficacy was demonstrated by Filatov's^18^ work and Pelaez's^19^ work first and from those of Meduri et al^20^ and Limoli^21,22^ later.

The authors describe functional stabilization in 60% of cases, functional improvement in 15% to 20% of cases, and worsening in 20% of cases. The clinical results, even though interesting, do not last, and after 6 to 18 months, according to the authors’ opinion and the type of patient being treated, degenerative disease and apoptotic processes linked to it resume their destructive action against the retina.

Other useful autologous sources are derivable from platelet-rich plasma (PRP) and adipose-derived stem cells (ADSCs) included in stromal vascular fractions (SVF) of adipose tissue.^23–27^

These elements, surgically grafted into suprachoroidal space, might delay retinal cell apoptosis through an almost pharmacological modulation of their secretions.^23–27^

Trophic effects of growth factors on retina are shown by the histological examination in guinea pig, considering the nuclear layer thickness of photoreceptors compared with untreated eye.

Therefore, clinical study is not possible on retina in humans. Moreover, human pathological variables are much more complex than those encountered in genetically modified animal models and treated under standard conditions.^5,28^

Moreover, similarly to the method of assessment used in animal models, we can use the electrophysiological testing also in the study of human retinal degeneration.^29,30^ In fact, these are noninvasive, objective, and reproducible examinations.

Schematically, for this study, we have divided the electroretinogram (ERG) into

1. scotopic rod-ERG that records the electrical response of the rods in the extrafoveal retinal areas;
2. scotopic maximal rod-cone-ERG that records the electrical response of the rods and cones of the entire retina;
3. photopic cone-ERG that records the electrical response of the cones in the foveal retinal area.

It represents a perfect indicator of cell viability, characterizes the quality of the cell electrical responses, and documents therapeutic effects during medical or surgical therapies.^31–34^

The aim of this work was to demonstrate, through the objectivity of the assessment electrofunctional test, a possible therapeutic effect of the joined suprachoroidal graft of adipocytes, ADSCs in SVF, and PRP, which, according to the cited literature, are able to produce growth factors.^14–17,23–27^ These factors, pouring in choroidal blood flow, could reach the retinal cells that are on the opposite side of the choroid, interacting directly with photoreceptors through their membrane receptors or indirectly through the interaction with other cells such as retinal pigment epithelial cells (RPEs), Müller cells, and endothelial cells.

## PATIENTS AND METHODS

*The study protocol was approved by the Ethical Committee*, Low Vision Research of Milan, and in accordance with Helsinki Declaration, written consent was obtained from all the patients.^35^

For this purpose, we have included in the study, 12 eyes of 12 patients affected from dry age-related macular degeneration (AMD). They were identified and enrolled according to several characteristics. The inclusion criteria were as follows:

1. participants of whites classified as well-nourished;
2. AMD diagnosis with spectral domain optical coherence tomography (SD-OCT), fundus autofluorescence imaging, and fluorescein angiography in the presence of drusen and irregularities of RPEs in at least 1 eye;
3. good storage extrafoveal areas;
4. measurable visual acuity;
5. normal intraocular pressure;
6. acceptance of the clinical protocol by signing the informed consent.

The exclusion criteria from the study for possible cross-related interference with the test were as follows:

1. patients with signs of exudative AMD;
2. myopia with spherical equivalent >6 diopters;
3. cataract, chorioretinal disorders, such as macular pucker or neovascular membrane-associated and other ocular disorders (glaucoma, optic neuritis, ocular trauma, high refractive errors, etc.);
4. insufficient compliance in individuals affected by medical problems, such as hypovitaminoses, multiple sclerosis, epilepsy, Parkinson disease, diabetes, hypertension, vasculitis, renal and hepatic diseases, gastrointestinal malabsorption, hypothyroidism, malignant neoplasias, and other systemic chronic diseases.

For each patient, the diagnosis was confirmed by confocal scanning laser ophthalmoscope Nidek F10 (Nidek Inc, Fremont, CA), SD-OCT Cirrus (Carl Zeiss Meditec AG, Jena, Germany), and microperimetry Maia 100809 (CenterVue S.p.A., Padova, Italy) or standard automated perimetry Octopus 900 (Haag-Streit AG, Koeniz, Switzerland).

Before the graft (T0), for each patient we evaluated best corrected visual acuity (BCVA) for far and near distance. BCVA was measured with early treatment diabetic retinopathy study charts at 4 m in logarithm of the minimum angle of resolution (logMAR) and visual acuity for near vision (close-up) in points (Pts). Therefore, we recorded the electrical cell activity through ERG (electromedical system of ocular electrophysiology, Retimax; C.S.O. Srl, Scandicci, Italy) according to the standards set in 2009 by the International Society for Clinical Electrophysiology of Vision.^30^

Each eye received a cell in graft between choroid and sclera called for convenience Limoli retinal restoration technique (LRRT), which is a variant of the intervention of Pelaez,^19^ who used a transplantation of autologous fat in subscleral space, obtaining interesting but transient results.^21–27^

In the LRRT variant, the following changes have been made with the purpose of increasing the survival of autologous fat graft,^36–38^ triggering the proliferation of ADSCs to promote the growth of choroidal perfusion, and obtaining a more complete modulation of the action of the factors secreted by the fat only.^39,25–27^

1. The distance between grafted autologous cells and choroid has been reduced by means of deep sclerectomy favoring the paracrine secretion of the autologous cells into the choroidal flow.
2. For the same reason, the area of contact between stalk and choroid has been expanded.
3. Suprachoroidal pocket has been built to accommodate the graft and saturated with a mixture of ADSCs and SVF obtained, according to the technique by Coleman et al^36^ and Lawrence et al.^37^
4. Adipose pedicle has been infiltrated with PRP gel obtained by centrifugation of the blood material, separation of the component, and its platelet degranulation.^23,24^

The details are as follows:

1. A deep scleral door of about 5 × 5 mm has been opened by a radial zip in the inferotemporal quadrant to 8 mm from the limbus. The sclerectomy must be deep enough to allow viewing of the slate color of the choroid.
2. Orbital fat is extracted from a gap above the inferior oblique muscle. The pedicle fat extracted must be sufficient to ensure the survival of vascular scaffold after its location.
3. The flap of autologous fat is laid gently on the bed and secured with choroidal vicryl 6/0 at the proximal edge of the door.
4. The scleral flap is then secured in order to avoid compression on the fat pedicle or on its nutrient vessels.
5. The remaining space between autologous fat graft, choroid, and scleral flaps are saturated with 0.5 cc. of SVF, previously prepared, full of ADSCs by venflon inserted into the scleral pocket.
6. Then, the stroma of the peduncle is infiltrated with 1 cc. of PRP using 25-gauge cannula.

Therefore, an autograft consisting of fat cells, ADSCs from SVF, and PRP has been realized.

A month after the LRRT (T30), we evaluated visual acuity and recorded cellular responses by scotopic, mesopic, and photopic ERG.

### Statistical Analysis

Data were entered into an Access database and then analyzed with R. After internal consistency checks, statistical analyses and data visualization were performed before and after surgery LRRT. In order to test if the differences pre- and post-treatment were significant, the Wilcoxon signed-rank test has been performed. The test is well suited when the differences between paired samples are not supposed to be normally distributed, as in this case. Then, the values of visual acuity, scotopic-ERG, and photopic-ERG were considered at the various stages of analysis, and the null hypothesis that there was no significant treatment effect was rejected if *P* < 0.05 or *P* < 0.1.

## RESULTS

We have included in the study, 12 eyes (7 right and 5 left eyes) of 12 patients (6 men and 6 women) with dry AMD, belonging to patients aged 71.25 years, between 62 and 80 years (SD ± 6.8).

**FIGURE 1:**
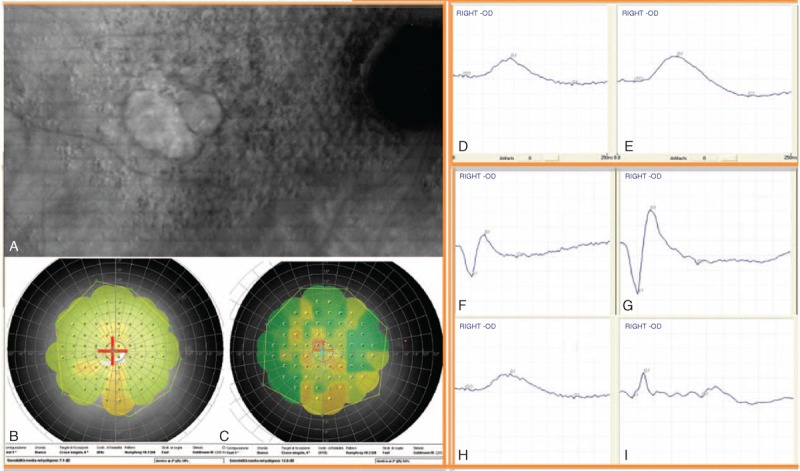
Panel (A) Patient affected from dry AMD. Panel (B–C) Microperimetric (increased from 7.1 to 12 dB) and ERG changes before and after 30 days of autograft cells by variant second Limoli. Scotopic rod-ERG (D–E); scotopic maximal rod-cone-ERG (F–G); photopic cone-ERG (H–I). AMD = age-related macular degeneration. ERG = electroretinogram.

The characteristics of the ocular functionality in surgery patients (Fig. 1) and the average values recorded before (T0) and after (T30) 30 days of autograft cells by variant second Limoli are shown in Tables 1 and 2. In any case, we reported adverse effects, and this highlights the nondangerousness of the procedure. Mean values recorded before (T0) and after (T30) surgery of the intraocular pressure were not changed significantly.

**TABLE 1:**
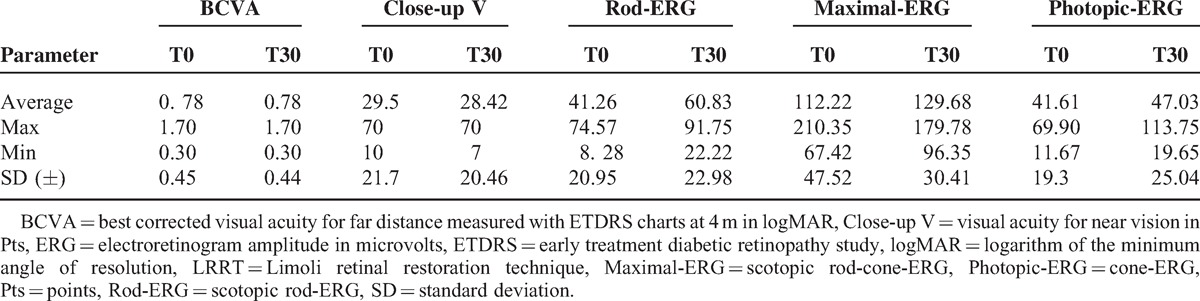
Mean Values Recorded Before (T0) and After (T30) Surgery With LRRT

**TABLE 2:**
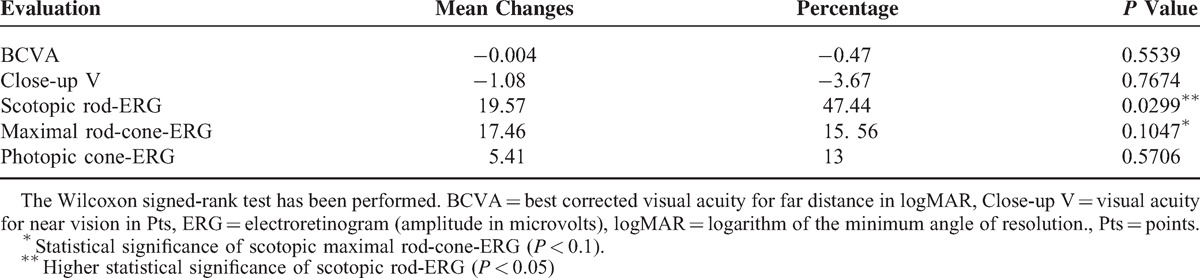
Mean Changes Pre- and Post-treatment and Statistical Significance of the Differences With Percentage Change of the Mean Values

**FIGURE 2:**
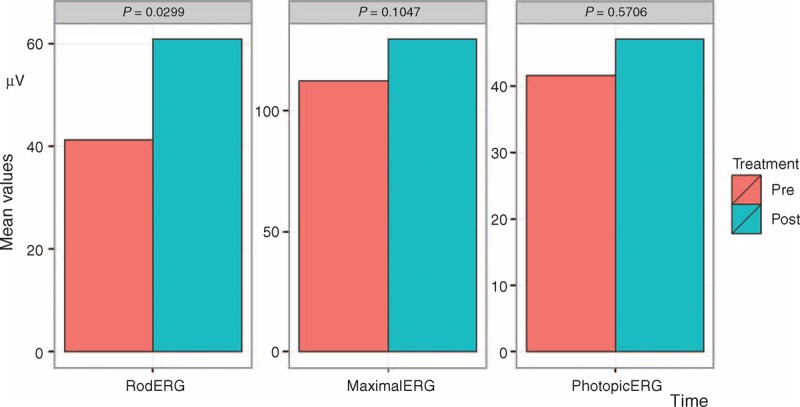
Electroretinographic changes. ERG before and after surgery with LRRT. Left Panel. Scotopic rod-ERG has increased from 41.26 to 60.83 μV with an average increase of 47.44%, significant at 5% level (*P* < 0.05). Central Panel. Scotopic maximal rod-cone-ERG has increased from 112.22 to 129.68 μV with an average increase of 15.56%, moderately significant at 10% level (*P* < 0.1). Right Panel. Photopic cone-ERG has increased from 41.61 to 47.03 μV with an average increase of 13%, not significant. ERG = electroretinogram, LRRT = Limoli retinal restoration technique.

**FIGURE 3:**
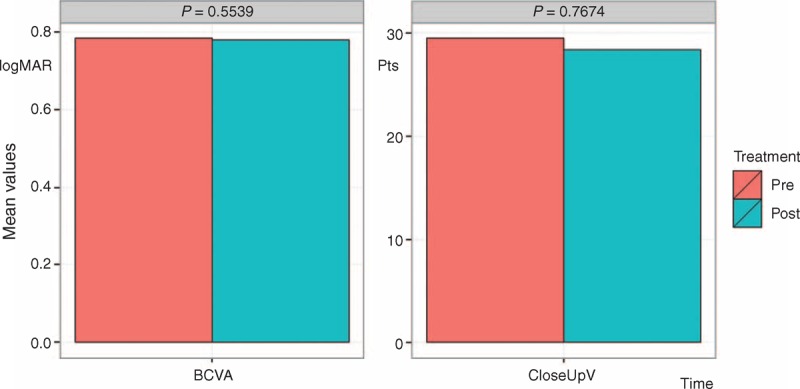
Visual acuity changes before and after surgery with LRRT were not statistically significant. Left Panel. BCVA for far distance in logMAR. Right Panel. Visual acuity for near in Pts. BCVA = best corrected visual acuity, logMAR = logarithm of the minimum angle of resolution, LRRT = Limoli retinal restoration technique, Pts = points.

The results after surgery with LRRT indicated the following:

1. electrofunctional response from the scotopic rod-ERG (Fig. 2, left panel) has increased from 41.26 to 60.83 μV with an average increase of 47.44%, significant at 5% level (*P* < 0.05);
2. electrofunctional response from the whole retina, scotopic maximal rod-cone-ERG (Fig. 2, central panel), has increased from 112.22 to 129.68 μV with an average increase of 15.56%, moderately significant at 10% level (*P* < 0.1);
3. electrofunctional answer coming from the photopic cone-ERG (Fig. 2, right panel) has increased from 41.61 to 47.03 μV with an average increase of 13%, not significant (*P* > 0.05);
4. 0.78 logMAR BCVA remains practically unchanged, therefore, with no significant change (Fig. 3, left panel);
5. Close-up has gone up from 29.5 to 28.42 Pts with variation of 3.67%, not statistically significant (Fig. 3, right panel).

## DISCUSSION

Therefore, the LRRT purpose, that is, the graft joint suprachoroidal adipose pedicle, of ADSCs in SVF, and intrapeduncolar PRP, was as follows:

1. promote the vascular pedicle fat engraftment with the underlying choroid;
2. enhance the pedicle fat original vascularization, in order to ensure its volume and survival;
3. give a regenerative start-up to all the retinal elements, through the secretion of paracrine growth factors.

As it is well known, the dry AMD shows a damage starting from foveal area, populated by cones, which typically has a reduced cellularity.

Extrafoveal areas, mainly populated by rods, typically appear better preserved.

Scotopic ERG is an outer retina function, so an increase of it implies an improvement in the ability to respond to stimulation by the photoreceptors.

LRRT (grafting of mature fat cells and ADSCs in SVF enriched with PRP) seems to have a positive effect on the increase in the various ERG values. This effect may be exercised due to the paracrine secretion properties of these cells, we hypothesize, including growth factors.

Consequently, retinal electrofunctional tests, that record full-field ERG responses, show different results after 30 days from the LRRT surgery technique. Unfortunately, in this pilot study, it was not possible to show data of the multifocal ERG, that records electrical responses from the different areas of the retina, because some patients had poor central fixation lack. Analogous difficulty was obtained for statistical analysis by microperimetry Maia and standard perimetry Octopus.

However, the most significant increase is recorded by scotopic ERG, whereas the less significant is the one recorded by photopic-ERG. The position of the graft near the choroid could allow at the product factors to flow into the choroidal flow, to reach receptors of endothelial cells, RPEs, Müller cells, photoreceptors, and potentially to interact with them. So, the mechanism of this effect may be due to the growth factors’ action on trophic photoreceptors introduced at the level of the retinal choriocapillaris through its contact with cell autologous graft. The foveal damage, mainly in the dry AMD, and the resulting reduced presence of cones at this level would explain the less significant increase of photopic ERG. Conversely, the best preservation of extrafoveal areas, and the consequent increased presence of rods in those areas, would explain the most significant increase of the scotopic ERG recorded response. In fact, growth factors need still viable cells, which possess the corresponding membrane receptors.

Following the complex growth factor receptor formation, a limited number of second messengers are generated within the cell target. These in turn control a series of biochemical pathways within the cell, by regulating the enzyme activity and transcription factors, through a series of cascade of phosphorylation events.^40–44^ These signals, arrived in the nucleus of the cell, determine changes in the response of genes, increasing proteins, enzymes, and cytokine transcription that play their role in cell trophic control.^40–44^

The main function of growth factors is the external control of the cell cycle, by the cell quiescence abandonment (G0 phase) and the cell entry in the G1 phase (growth). However, this is not their only function; they can regulate the entry into mitosis, cell survival, migration, and cell differentiation.^40,41^

The LRRT allows the grafting of 3 autologous components and each of the grafted elements has its own specific assets in growth factors useful from a point of view regenerative:

1. Fat cells, contained in the pedicle placed in the suprachoroidal space, produce basic fibroblast growth factor (bFGF), epidermal growth factor (EGF), insulin-like growth factor-1 (IGF-1), interleukin (IL), transforming growth factor-β (TGF-β), pigment-epithelium-derived factor (PEDF), and adiponectin.^14–17^
2. The ADSCs produce bFGF, vascular endothelial growth factor (VEGF), macrophage colony-stimulating factor (M-CSF), granulocyte-macrophage colony-stimulating factor (GM-CSF), placental growth factor (PlGF), TGF-β, hepatocyte growth factor, IGF-1, IL, and angiogenin.^25–27,44^
3. Platelets produce platelet-derived growth factor (PDGF), IGF-1, TGF-β, VEGF, bFGF, EGF, platelet-derived angiogenesis factor (PDAF), and thrombospondin (TSP).^23,24^

Some factors, such as VEGF, bFGF, angiogenin, PDAF, PlGF, PDGF, EGF, and TGF- β, promote endothelial regeneration and may contribute to the choriocapillaris reperfusion.^42,43,45^ VEGF introduced with the PRP stimulates ADSCs proliferation that in turn promote both the autologous fat and adipocytes survival.^38,39,42^

Others, such as the TSP and the PEDF inhibit neovascular processes;^46–48^ bFGF acts directly on the photoreceptor, binding to the surface receptors and promotes their survival.^46–48,10,49^ The PEDF similarly to bFGF has neurotrophic activity in respect of the photoreceptors.^50^

Some factors, however, as the EGF, act on Müller cells inducing endogenous bFGF transcription and strengthen the protective action of these cells toward the retina nerve cells.^51–53^ EGF also stimulates the ADSCs to increase its secretory activity.^54^

Furthermore, bFGF, IGF-1, PEDF, PDGF, TGF-β, and VEGF are normally secreted by RPEs, which in the presence of maculopathy to atrophic evolution are pathologically deficient for the involution of RPEs/choriocapillaris system. Their paracrine secretion by cell graft helps to promote the photoreceptor and choriocapillaris survival.^55–59^

Moreover, M-CSF, GM-CSF, and IL have an anti-inflammatory component and a chemotactic one toward macrophages that contribute to remove cell intraretinal debris and function normally carried out by RPEs.^60–63^ Also from an experimental point of view, increases in the number of macrophages have been observed after intravitreal injection of bFGF.^11,44^

From the results of our study, with LRRT technique it seems possible to achieve, directly and indirectly, an increase in choroidal perfusion and a greater tropism of the photoreceptors through bFGF–receptor interactions and stimulation mediated by Müller cells, RPEs, and retinal photoreceptors. Successively, these events would allow a growth of the cellular activity to facilitate appropriate potentials of action.

It is possible affirm that an improved cell trophism translates into an electrical activity increase of the cell, which can be measured objectively through the classic electrofunctional testing as evidenced in our results. In the near future, we will continue the researches by the enrollment of the largest number subjects with more visual acuity and central fixed, through the statistical valuation of all the indispensable examinations to confirm technique validity and study of biochemical effects.

## CONCLUSION

From a clinical point of view, cell-mediated therapy based on the use of growth factors appears interesting because they can improve the electrical cell responses in the short term. The ERG could, therefore, be used to monitor in an objective manner in humans, similar to the guinea pigs, the effect of regenerative cellular-mediated therapies, including autologous fat orbital joint to cells derived from ADSCs in adipose tissue SVF and PRP (LRRT). It is possible to assume a greater effect even at foveal level provided that it is applied early, such as to allow a broader cytokine–receptor interaction, when most of the photoreceptors are still present. Therefore, we have to take into account that the cell-mediated administration of growth factors can be realized by using a scleral surgery.

In summary, it seems essential in the future to investigate the long-term effectiveness of this therapy, the cost-benefit ratio of the intervention, the best time of intervention, and the real security for the future visual acuity of our patients.
